# Physical Activity and Mental Health of Employed Adults: Mediation and Moderation Effects of Beliefs in the Benefits of Physical Activity

**DOI:** 10.3390/ijerph21070854

**Published:** 2024-06-29

**Authors:** Dragan Glavaš, Irena Pavela Banai

**Affiliations:** 1 Department of Psychology, Catholic University of Croatia, 10 000 Zagreb, Croatia; dragan.glavas@unicath.hr; 2 Department of Psychology, Faculty of Humanities and Social Sciences, Josip Juraj Strossmayer University of Osijek, 31 000 Osijek, Croatia

**Keywords:** physical activity, mental health, depression, anxiety

## Abstract

Numerous studies have shown physical activity (PA) improves psychological functioning and well-being. However, the underpinning processes and mediating variables are less known. There is evidence that beliefs about the benefits of PA contribute to physical health, regardless of actual PA. By applying these findings in the context of mental health, we sought to investigate the role of belief in the benefits of PA in the relationship between self-reported PA and mental health. A total of 381 employed adults completed the Godin Leisure-Time Exercise Questionnaire, Center for Epidemiologic Studies Depression Scale, and Anxiety subscale of the Emotional state scale. Furthermore, participants reported the degree to which they believe that PA has benefits for their psychological health. The mediation analysis shows that greater PA intensity was associated with lower levels of depression and anxiety. Additionally, belief in the benefits of PA on psychological health had a mediating role. Specifically, more intensive PA was related to a stronger belief in PA benefits, subsequently leading to reduced levels of depression and anxiety. We discuss the neurobiological mechanisms underlying the relationship between PA and mental health, alongside the significant role of mindset.

## 1. Introduction

The benefits of physical activity (PA) on physical health are well-known and widespread. Consistently, research shows that regular PA helps prevent a wide range of chronic diseases, improves various health outcomes, and reduces mortality rates (for a review, see [1]).

Although the physical benefits of PA are widely recognized, the mental health benefits are equally considerable and deserve further scrutiny. Numerous studies have confirmed the positive effects of PA on various aspects of psychological functioning and psychological well-being.

Psychological well-being refers to the sum of feeling good and functioning effectively [2]. It has been suggested that intentional activities, that is, activities over which we have control, are important drivers of psychological well-being [3,4].

One such intentional activity that may significantly contribute to psychological well-being is physical activity. Here, we focus on PA as an intentional behavior but also on the role of the cognitive interpretation of the positive effect of PA on psychological health and their relationship to self-reported depression and anxiety.

### 1.1. Physical Activity and Mental Health

In this context, it has been reported that regular exercise is associated with significant improvements in general well-being, particularly in mood, sense of coherence, fortitude, stress, and coping [5], and with an enhanced health-related quality of life [6] among the adult population. Similarly, research also shows that regular PA is associated with life satisfaction [7], psychological well-being, positive relations, and a sense of purpose in life [8] among students. This potential positive effect of PA is supported by experimental data. Namely, Klaperski and Fuchs [9] randomly assigned healthy and insufficiently active men to a 12-week training program and found that exercise, just like a relaxation training program, buffered the negative effects of stress on general and mental health. The robustness of these findings is further emphasized by the fact that physical fitness did not moderate the observed impact of physical activity.

Similarly, numerous studies have investigated the role of regular PA in the context of mental health and psychological difficulties related to depression and anxiety. For example, previous studies investigating the effects of physical activities on depression, anxiety, and psychological distress suggest that PA may have similar beneficial effects to psychotherapy and pharmacotherapy. Specifically, Kvam et al. [10] concluded that physical exercise is an effective intervention for depression, and it can be an adequate additional treatment in addition to antidepressants. Furthermore, it has been reported that individuals who engage in any type of PA have lower odds of experiencing mental health problems in comparison to inactive individuals [11]. Likewise, it has been shown that PA levels (moderate and high) were negatively related to self-reported anxiety and depression symptoms [12].

Additionally, it has been suggested that PA protects from agoraphobia and post-traumatic disorder [13]. Similarly, a meta-analysis investigated the relationship between PA and mental health during the COVID-19 pandemic. The results suggest that higher PA was related to higher well-being and quality of life. On the other hand, a negative relationship was found between PA and depressive symptoms, anxiety, and stress [14]. Hence, the authors concluded that PA is an effective way to mitigate the adverse effects of the COVID-19 pandemic on mental health. Considering the data provided by the World Health Organisation (WHO), which indicate a 25% increase in the prevalence of anxiety and depression globally due to the COVID-19 pandemic, this finding has significant implications for public health policies. It suggests that promoting physical activities during global social and health crises, such as pandemics, could be beneficial.

Finally, a recent synthesis of the evidence on the effects of PA on depression, anxiety, and psychological distress in adult populations, which considered randomized controlled trials, reveals the benefits of PA on symptoms of depression, anxiety, and distress across a wide range of adult population (the general population, people with diagnosed mental health disorders, and people with chronic disease) [15].

By arguing that focus should be directed toward the non-clinical population and the potential mental health benefits of physical activity, Rebar et al. [16] reported that PA reduces depression and anxiety in non-clinical populations as well.

Together, these findings strongly suggest that PA may serve as a successful preventive mechanism in the context of mental health, particularly under the current circumstances. Hence, in raising public awareness of mental health issues and emphasizing prevention that might be more critical now than ever, we should underscore the significance and advantages of engaging in physical activity. Nevertheless, despite empirical evidence indicating the direct beneficial effects of PA on mental health, it is important to understand the underlying mechanisms and the potential role of other factors in this effect. Beyond the direct effects of PA, the evidence suggests that individuals’ beliefs about the benefits of PA (i.e., mindset) may significantly impact their health outcomes.

### 1.2. Mindset, Physical Activity, and Health Outcomes

Individuals’ beliefs about the benefits of PA may be of particular interest when considering a finding that emerged in investigating the association between PA and physical health.

Specifically, in their seminal work, Crum and Langer [17] found that beliefs and mindset affect the benefits of physical activity. In their study, the experimental group of hotel room attendants was told that their regular employment activities meet active living guidelines. The control group did not receive any information. Four weeks following the intervention, the experimental group attendants showed lower weight, blood pressure, body fat, waist-to-hip ratio, and BMI. In contrast, the control group showed no changes. A shift in mindset, rather than an actual increase in activity during the intervention, appears to have caused this improvement in physical health indices.

Since then, a growing body of research suggests that mindsets about various aspects of health-relevant behaviors play a role in health and well-being outcomes. In a recent study, Zahrt et al. [18] randomly assigned participants into four groups to study mindsets about PA adequacy and health benefits. Each group received feedback about their daily step count via wearable fitness trackers: either accurate, inflated, deflated step counts or accurate step counts, along with a mindset intervention to encourage positive activity adequacy mindsets. It was found that participants in the fourth group reported improved effects, functional health, and self-reported physical activity compared to participants receiving accurate step counts only. These findings imply that people have mindsets about their PA level and, most notably for this study, beliefs or knowledge about its efficacy. Their beliefs likely affected their physical health, regardless of their actual physical activity.

### 1.3. The Present Study

By applying these findings and shifting the focus to mental health outcomes, the aim of this study was to explore whether beliefs in the benefits of PA moderate or mediate the relationship between PA and mental health outcomes, specifically depression and anxiety, in a non-clinical sample of young adults. Based on previous research, we hypothesized that the intensity of PA would be negatively related to self-reported anxiety and depression issues symptoms, and these relationships would be mediated by the degree to which participants believe in the beneficial effects of their physical activity.

## 2. Materials and Methods

### 2.1. Participants

This study encompassed a convenient sample of 381 employed adults in Croatia (64.53% were female) aged between 21 and 42 years (M = 32.01, SD = 5.34) who participated in the research project “Physical activity and psychological well-being: from habit to identity”. The sample consisted of the participants who responded to an invitation letter that encompassed the research project details and the instruction that the study targets all healthy individuals up to their early forties. An online questionnaire hyperlink was distributed by email, smartphone applications, and social media platforms. Most participants finished graduate study (56.4%), whereas 14.2% finished undergraduate study, 12.9% completed postgraduate study, and 16.5% finished high school.

Due to incomplete responses, seven participants were excluded from the final analysis.

### 2.2. Instruments

#### 2.2.1. Physical Activity Intensity

For assessing the physical activity intensity, we used The Godin-Shephard Leisure-Time Physical Activity Questionnaire (GSLTPAQ) [19] translated into Croatian using the back translation. Studies supporting the validity of this scale to distinguish between less and more physically active people included those that demonstrate the relationship between GSLTPAQ results and physical fitness indicators [20], as well as the differences in physical fitness indicators between active and insufficiently active, as classified by the Godin-Shepard Leisure-Time Physical Activity Questionnaire [21].

To determine how often participants engaged in PA during a typical week, participants were asked to answer the question, “During a typical 7-day period (week), how many times, on average, do you do the following kind of exercise for more than 15 min?”. Participants responded how many times during a typical week they engaged in strenuous (heart beats rapidly, e.g., running, jogging, football, etc.), moderate (not exhausting, e.g., fast walking, tennis, etc.), and mild (minimal effort, e.g., yoga, bowling, etc.) PA. The overall score was calculated by multiplying each individual score by its corresponding ponder (3 for mild exercise, 5 for moderate exercise, and 9 for strenuous exercise) and then summing the multiplication results. A higher score indicates a more prominent intensity of physical activity.

#### 2.2.2. Anxiety

To assess anxiety, we used the Anxiety subscale of the Emotional state scale [22]. The scale was developed to circumvent challenges encountered when converting scales from a foreign language, namely, difficulties arising from the translation of particular adjectives [22]. The scale shows good psychometric properties [23,24]. It comprises eight items (e.g., “worried”) that reflect one’s feelings. The extent to which participants identified with the specified items was assessed using a 5-point scale ranging from 1 (not at all or very little) to 5 (very strongly or entirely). A total score was obtained as a linear composite of eight items, with higher scores indicating more intense anxiety symptoms. The reliability of the anxiety subscale in this study, as measured with the internal consistency coefficient, suggested highly satisfactory reliability (Cronbach α = 0.94).

#### 2.2.3. Depression

We used a 10-item version of the Center for Epidemiologic Studies Depression Scale (CES-D) [25] to assess depression in the past week. The 10-item version is a shortened version of the revised original 20-item scale, which has demonstrated its validity and reliability as a measure of depression in healthy adults [26,27]. The 10-item CES-D version has also been demonstrated to be a valid and reliable measure of depression, even though there is inconsistent empirical evidence on the potential removal of positive items [28].

Participants rated each item (e.g., “My sleep was restless”) on a 4-point scale ranging from 0 (rarely or none of the time (less than one day)) to 3 (most of the time (5–7 days)). A total score was obtained as a linear composite of all items. Higher scores indicated a greater severity of depression symptoms. The value of Cronbach α = 0.83 indicated good internal consistency and reliability of the scale.

#### 2.2.4. Belief in the Positive Effect of PA on Psychological Health

The participants’ belief in the beneficial effect of PA on their psychological health was evaluated using the following question, designed explicitly for the purpose of the present study: “Do you think that the amount of your physical activity has a positive effect on your psychological health?”. Participants responded on a 7-point scale, ranging from 1 (no, not at all) to 7 (yes, extremely).

### 2.3. Procedure

The data were collected online as part of a broader research project titled “Physical Activity and Psychological Well-being: From Habit to Identity”, funded by the Catholic University of Croatia and approved by the Ethics Committee of the Catholic University of Croatia (Document Class: 641-03/22-03/10; No.: 498-15-06-22-005).

The project survey was conducted using the web platform SurveyRock and was active for three weeks in April 2022. The invitation letter was disseminated by the team of research project associates and partner institutions with the aim of reaching all regions of the country. Participation in the project was voluntary. However, the research team offered participants individual insights into the project’s scientific goals, practical applications, and counselling on PA and mental health.

Before participating in the survey, participants provided informed consent on a form outlining the study’s objectives, the confidentiality and anonymity of the data, and the possibility of withdrawing from the study without any consequences. In addition to the survey design, which ensures the security and anonymity of the data, we adjusted the settings of the web platform to prevent the storage of IP addresses. Participants consented to participate in the study by tapping the “next” option and proceeding to the survey.

### 2.4. Statistical Analysis

For the statistical analysis, first, we depicted the descriptive statistics (average, standard deviation, and range) of the tested variables. To investigate the relationship between the variables, we used the Pearson correlation coefficient. To test the direct and indirect contribution of PA intensity on reported anxiety and depression and investigate the mediation and moderation role of belief in the beneficial effect of PA on psychological health in this relationship, we utilized mediation and moderation analysis using the PROCESS macro (path analysis modelling tool) for SPSS [29].

The regression coefficients (*b*s) of the tested mediation models estimated the predictive role of PA intensity in explaining anxiety and depression scores, while the belief in the beneficial effect of PA on psychological health was entered as a mediator. Simply put, the mediation models showed the direct contribution of PA intensity to anxiety and depression and the indirect contribution of PA intensity to the anxiety and depression scores, mediated by belief in the beneficial effect of PA on psychological health.

On the other hand, in the moderation models, belief in the beneficial impact of PA on psychological health was entered as a moderator. Specifically, we tested the predictive role of PA intensity in explaining the anxiety and depression scores and the dependence of this prediction on belief in the beneficial impact of PA on psychological health, i.e., the moderation role of belief in the beneficial impact of PA.

## 3. Results

### 3.1. Data Analysis

We tested the direct and indirect contribution of PA intensity on reported anxiety and depression and the mediation and moderation role of belief in the beneficial impact of PA on psychological health in this relationship, utilizing mediation and moderation analysis using a PROCESS macro [29].

### 3.2. Descriptive Statistics and Correlation Analysis

Table 1 shows the descriptive statistics for PA intensity, belief in the beneficial effect of PA on psychological health, and self-reported anxiety and depression.

The correlation analyses (Table 1) reveal a significant weak negative relationship between PA intensity and anxiety (r = −19, *p* < 0.01) and depression (r = −20, *p* < 0.01). Belief in the beneficial effect of PA on psychological health was also negatively correlated with anxiety (r = −0.25, *p* < 0.01) and depression (r = −0.17, *p* < 0.01). Furthermore, a positive association was found between PA intensity and belief in the beneficial effect of PA on psychological health.

Therefore, as expected, participants who reported more intensity of PA during a typical week showed lower levels of anxiety and depression. Likewise, participants who reported a stronger belief in the beneficial effects of PA reported lower levels of psychological symptoms.

Next, we sought to investigate the mediation and moderation role of belief in the beneficial effect of PA in the found relationship between PA intensity and psychological symptoms.

### 3.3. Mediation and Moderation Analysis

To determine the mediation and moderation roles of the mentioned belief, the mediation and moderation analyses using the PROCESS macro were conducted separately for the relationship between PA intensity and two mental health variables: anxiety and depression.

**Figure 1:**
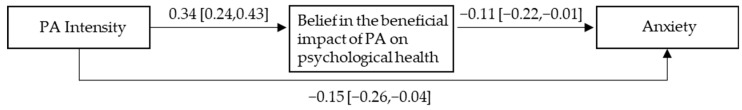
The mediating role of belief in the beneficial effect of physical activity on psychological health on the relationship between PA intensity and anxiety.

The model showing the relationship between the belief in the beneficial effect of physical activity, PA intensity, and anxiety is presented in Figure 1.

As expected, the mediation analysis reveals a significant negative direct effect of PA intensity on anxiety, suggesting that increased PA intensity predicts lower levels of anxiety (*b* = −0.15 [−0.26,−0.04]). In addition, the indirect effect of PA intensity on anxiety, mediated by the belief in the beneficial effect of PA on psychological health, was also statistically significant (*b* = −0.04 [−0.08,−0.01]). Specifically, PA intensity was positively related to belief in the beneficial effect of PA, which was, in turn, negatively associated with anxiety.

The overall mediation model accounted for 4.69% of anxiety scores (F(2,371) = 9.12, *p* < 0.001) (Figure 1).

Moreover, the moderation analysis was also conducted, with belief in the beneficial effect of PA as the moderator variable. However, the analysis reveals no significant moderator effect in the relationship between PA intensity and anxiety (*b* = −0.05 [−0.16,0.05]).

**Figure 2:**
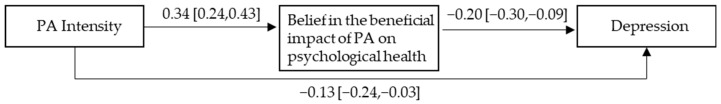
The mediating role of belief in the beneficial effect of physical activity on psychological health on the relationship between PA intensity and depression.

Next, we repeated the mediation analysis to investigate the mediating role of belief in the beneficial effect of PA on psychological health in the relationship between PA intensity and depression (Figure 2).

As predicted, a significant negative direct effect of PA intensity on self-reported depression was found, indicating that increased PA intensity predicts lower levels of depression symptoms (*b* = −0.13 [−0.24,−0.03]). Furthermore, the indirect effect of PA intensity on depression, mediated by the belief in the beneficial effect of PA on psychological health, was also statistically significant (*b* = −0.07 [−0.13,−0.02]). As in the case of anxiety, PA intensity was positively related to belief in the beneficial effect of PA, which was, in turn, negatively related to depression.

The overall mediation model explained 7.6% of the variance in depression scores (F(2,371) = 15.14, *p* < 0.001) (Figure 2).

Moreover, in line with the moderation analysis conducted for the anxiety scores, the moderating role of the belief in the beneficial effect of PA was not significant in the relationship between PA intensity and depression (*b* = −0.01 [−0.11,0.10]).

## 4. Discussion

The present study was motivated by the original findings regarding the benefits of physical activity (PA) on physical outcomes, suggesting that beliefs or knowledge about the efficacy of PA contribute to overall physical health [17,18]. In the present research, we aimed to investigate whether a similar effect exists when it comes to the association between PA and mental health. Based on previous findings [11,14,15], we assumed that PA would be negatively related to the self-reported levels of depression and anxiety, and these relationships would be mediated by the degree to which participants believe in the beneficial effects of their physical activity.

First, as predicted, increased PA intensity was associated with lower levels of both anxiety and depression. Participants who reported more frequent engagement in PA during a typical week experienced fewer mental health issues. Therefore, the present results support the notion emphasized by Bafageeh and Loux [11] that engaging in any type of PA might lower the odds of experiencing mental health issues. Similarly, the present results are in line with previous studies indicating negative relationships between PA levels (moderate and high) and self-reported anxiety and depression symptoms [15], as well as stress [14]. It is noteworthy, however, that the present study did not consider the specific type of PA. This aspect is important since previous studies have shown that only outdoor walking/running, cycling, and team sports were associated with lower odds of depression [11].

Moreover, the current study adds to the existing literature by showing a significant relationship between PA and mental health in a non-clinical sample of employed adults. This result complements previous findings showing that the largest beneficial effects of PA were observed in people with depression, HIV, and some chronic diseases (e.g., kidney disease), as well as in pregnant and postpartum women [15].

The previous and present findings, therefore, suggest that PA has benefits that can be used in promoting mental health and mental health awareness. Hence, the practical implications of these findings are clear. The implementation of PA and promotion of the benefits of physical activity, not just for physical but also for mental health and psychological well-being, should become a vital strategy within the health support system and public health policies.

From a neurobiological perspective, the results of this study provide further support to the possible neurobiological mechanisms involved in the anxiety-reducing and antidepressant effects of PA. Several neurobiological explanations exist to account for a variety of observed mental health benefits of PA. One such mechanism is the release of monoamine neurotransmitters in the brain by the increased physical activity (i.e., serotonin, dopamine, and norepinephrine) (for a review, see [30,31]). The elevation of these neurotransmitters acts as a treatment for their imbalance, which is associated with symptoms of depression [32]. Another system that can be induced by increased PA is the endocannabinoid system. This system, closely related to the dopamine system, among other roles, is essential in regulating stress, emotions, and well-being. Extensive studies have been conducted on the hypothesis of endogenous endocannabinoids as the genuine origin of pain relief, sedation, anxiety reduction, and decreased depression in individuals who exercise (for a review, see Matei et al. [33]). Engaging in PA can also result in the activation of the brain-derived neurotrophic factor (BDNF), which, in addition to its mediation role in the connection between exercise and the growth of new neurons in the hippocampus [34], regulates stress and anxiety-related behaviors [35]. While progress has been made in comprehending the neurobiological mechanisms underlying the positive effect of PA, further research is needed to develop a comprehensive neurobiological model that can elucidate the antidepressant, anxiolytic, and other advantageous effects of PA.

Second, this study confirms and expands upon what we know about the relationship between PA and mental health. Specifically, we addressed further research questions regarding the role of belief in the beneficial effect of PA on psychological health. As hypothesized, the extent to which participants believe that their PA has a beneficial effect on their psychological health was found to mediate the relationship between PA intensity and self-reported levels of anxiety and depression. The present study reveals that PA intensity was positively related to belief in the beneficial effect of PA, which was, in turn, negatively associated with self-reported levels of anxiety and depression. Having in mind the limitations of correlational design, we can only speculate that PA intensity decreased anxiety and depression levels by strengthening belief in the beneficial effect of PA on mental health. In any case, the presented findings confirm the significant role of beliefs.

A significant effect of belief in the benefits of PA in the context of reducing mental health issues may be related to the potential role of psychological mindsets as core assumptions about specific domains or categories. Zahrt et al. [18] noted that mindsets are often overlooked factors that can influence one’s health-relevant behaviors and processes. Having this in mind, the authors showed that one’s beliefs about the efficacy of their PA affected their physical health outcomes. For example, even when some participants objectively engage in the same amount of PA, those who believe that their activity level is beneficial and adequate may have greater benefits in terms of health outcomes. On the other hand, those who believe their PA is inadequate may experience worse health outcomes. Specifically, it has been shown that the perceived levels of PA predicted mortality, even after accounting for the effects of objectively measured PA [36].

Implementing these findings in the field of mental health outcomes, it is reasonable to assume that similar processes and mindset effects also took place in the present study. Regarding the exact mechanism through which mindset shapes mental health outcomes, affective processes might be of great importance. Specifically, beliefs that PA helps and benefits mental health may be related to positive emotional states, less stress, and greater well-being. In contrast, not believing in the beneficial effects of PA may be related to negative emotional states and, consequently, elevated psychological distress. This assumption may be addressed in future studies by assessing beliefs in the benefits of PA as well as changes in affective states when potentially manipulating beliefs. Therefore, future studies are necessary to validate the assumptions mentioned above that can be drawn from the present work.

With this in mind, additional studies are required to understand the key tenets of the underlying mechanisms. Therefore, future studies should include experimental research trials to investigate the exact causal mechanism underpinning the relationships found in the present study. Moreover, planning and conducting specific interventions to increase PA, on the one hand, and to make beliefs about the benefits of PA stronger, on the other hand, may be a suitable experimental approach for future studies to address the limitation of the present correlational design. Moreover, there are some potentially open questions about the validity of the measure used to assess the belief in the beneficial effects of PA. Therefore, another promising line of research would be directed at developing new instruments to fully capture the role of beliefs. Another limitation is related to the lack of data regarding the specific types of physical activities participants engaged in. While the positive relationship between PA and mental health has been consistently supported in previous studies [14,15], less is known about how different types of activity affect mental health. There is, however, some evidence suggesting that running, sports, and weightlifting may have the greatest benefits when it comes to mental health [11]. Nevertheless, it has also been reported that resistance training and yoga have beneficial effects [15]. Future research should certainly test this finding further in the context of the role of mindset and psychological well-being. Another variable worth considering when discussing the benefits of PA and specific interventions is the duration of the intervention. It has been shown that longer interventions were less effective than shorter interventions, which may seem counterintuitive [15].

Generally, this line of research is of great importance because the previous and present research strongly supports the notion that PA has beneficial effects on depression, anxiety, and psychological distress, which may suggest its role in psychotherapy and pharmacotherapy. Still, as pointed out by Singh et al. [15], the benefits of PA are often overlooked in the management of mental health issues and psychological difficulties, such as depression and anxiety, in formal contexts and healthcare institutions. Therefore, we suggest that promoting and communicating the present findings to healthcare professionals and practitioners may be beneficial in terms of implementing these results in the mental healthcare system, especially since the present study shows that mindset also plays a role in the relationship between PA and mental health, meaning that if someone believes that PA has benefits, those beliefs may be effective in improving depression and anxiety across a broad range of populations. Increasing public awareness of the benefits of PA in this context may, in fact, lead to stronger beliefs and greater knowledge that PA actually helps in psychological well-being. As the present study shows, stronger beliefs were associated with greater psychological well-being and lower levels of depression and anxiety. Hence, the adequate communication and dissemination of the present findings to the general public and patients with mental health disorders is of great importance.

## 5. Conclusions

The presented findings are valuable in light of the relationship between PA and mental health. The present study reveals that the intensity of PA relates to lower levels of self-reported mental health problems and symptoms, with beliefs potentially having a significant role in this relationship. Whichever mechanisms underpin these relationships, it is important to point out the implications of the present findings. That is, the results allow the conclusion that one’s beliefs about the benefits of PA positively relate to psychological well-being. Therefore, raising awareness about these effects and making these research results available and suitable for the general public is of great importance. On this basis, we conclude that it is the responsibility of researchers, public health professionals, and practitioners to emphasize the role of PA and beliefs in its positive effects when it comes to promoting mental health awareness.

## Figures and Tables

**Table 1:** Descriptive statistics and intercorrelation between PA intensity, belief in the beneficial effect of PA on psychological health, and psychological symptoms (anxiety and depression).

	*M (SD)*	*Range*	1	2	3	4
PA intensity (1)	43.62 (24.37)	0–136	-	0.34 *	−0.19 *	−0.20 *
Belief in the beneficial effect of PA (2)	6.23 (1.30)	1–7		-	−0.25 *	−0.17 *
Anxiety (3)	17.83 (7.43)	8–40			-	0.72 *
Depression (4)	17.19 (5.19)	10–37				-

*Note:* * *p* < 0.01.

## Data Availability

The data supporting reported results can be found as publicly archived datasets generated during the study at: https://osf.io/ybdca/files/osfstorage (accessed on 26 June 2024).

## References

[1] Posadzki P., Pieper D., Bajpai R., Makaruk H., Könsgen N., Neuhaus A.L., Semwal M. (2020). Exercise/physical activity and health outcomes: An overview of Cochrane systematic reviews. BMC Public Health.

[2] Huppert F.A. (2009). Psychological well-being: Evidence regarding its causes and consequences. Appl. Psychol. Health Well-Being.

[3] Lyubomirsky S., Dickerhoof R., Worell J., Goodheart C.D. (2005). Subjective well-being. Handbook of Girls’ and Women’s Psychological Health: Gender and Wellbeing across the Life Span.

[4] Sheldon K.M., Lyubomirsky S. (2006). How to increase and sustain positive emotion: The effects of expressing gratitude and visualizing best possible selves. J. Posit. Psychol..

[5] Edwards S. (2006). Physical exercise and psychological well-being. S. Afr. J. Psychol..

[6] Bize R., Johnson J.A., Plotnikoff R.C. (2007). Physical activity level and health-related quality of life in the general adult population: A systematic review. Prev. Med..

[7] Liu F., Zhu Z., Jiang B. (2021). The influence of Chinese college students’ physical exercise on life satisfaction: The chain mediation effect of Core self-evaluation and positive emotion. Front. Psychol..

[8] Ugwueze F.C., Agbaje O.S., Umoke P.C.I., Ozoemena E.L. (2021). Relationship between Physical Activity Levels and Psychological Well-Being among Male University Students in South East, Nigeria: A Cross-Sectional Study. Am. J. Men’s Health.

[9] Klaperski S., Fuchs R. (2021). Investigation of the stress-buffering effect of physical exercise and fitness on mental and physical health outcomes in insufficiently active men: A randomized controlled trial. Ment. Health Phys. Act..

[10] Kvam S., Kleppe C.L., Nordhus I.H., Hovland A. (2016). Exercise as a treatment for depression: A meta-analysis. J. Affect. Disord..

[11] Bafageeh F., Loux T. (2022). The relationship between types of physical activity and mental health among US adults. Ment. Health Prev..

[12] Ghrouz A.K., Noohu M.M., Dilshad Manzar M., Warren Spence D., BaHammam A.S., Pandi-Perumal S.R. (2019). Physical activity and sleep quality in relation to mental health among college students. Sleep Breath..

[13] Schuch F.B., Stubbs B., Meyer J., Heissel A., Zech P., Vancampfort D., Rosenbaum S., Deenik J., Firth J., Ward P.B. (2019). Physical activity protects from incident anxiety: A meta-analysis of prospective cohort studies. Depress. Anxiety.

[14] Marconcin P., Werneck A.O., Peralta M., Ihle A., Gouveia É.R., Ferrari G., Sarmento H., Marques A. (2022). The association between physical activity and mental health during the first year of the COVID-19 pandemic: A systematic review. BMC Public Health.

[15] Singh B., Olds T., Curtis R., Dumuid D., Virgara R., Watson A., Szeto K., O’Connor E., Ferguson T., Eglitis E. (2023). Effectiveness of physical activity interventions for improving depression, anxiety and distress: An overview of systematic reviews. Br. J. Sports Med..

[16] Rebar A.L., Stanton R., Geard D., Short C., Duncan M.J., Vandelanotte C. (2015). A meta-meta-analysis of the effect of physical activity on depression and anxiety in non-clinical adult populations. Health Psychol. Rev..

[17] Crum A.J., Langer E.J. (2007). Mind-set matters: Exercise and the placebo effect. Psychol. Sci..

[18] Zahrt O.H., Evans K., Murnane E., Santoro E., Baiocchi M., Landay J., Delp S., Crum A. (2023). Effects of wearable fitness trackers and activity adequacy mindsets on affect, behavior, and health: Longitudinal randomized controlled trial. J. Med. Internet Res..

[19] Godin G. (2011). The Godin-Shephard Leisure-Time Physical Activity Questionnaire. Health Fit. J. Can..

[20] Miller D.J., Freedson P.S., Kline G.M. (1994). Comparison of activity levels using the Caltrac accelerometer and five questionnaires. Med. Sci. Sports Exerc..

[21] Amireault S., Godin G. (2015). The Godin-Shephard Leisure-Time Physical Activity Questionnaire: Validity Evidence Supporting its Use for Classifying Healthy Adults into Active and Insufficiently Active Categories. Percept. Mot. Ski..

[22] Sorić I., Lacković-Grgin K., Proroković A., Ćubela V., Penezić Z. (2002). Skala za mjerenje trenutnog emocionalnog stanja [The Emotional Experience Scale]. Zbirka Psihologijskih Skala i Upitnika—Svezak 1 [Collection of Psychological Scales and Questionnaires—Volume 1].

[23] Sorić I. (1998). Usporedba Osnovnih Postavki Weinerove i Lazarusove Teorije Emocija u Školskoj Situaciji Ispitivanja Znanja. Ph.D. Thesis.

[24] Petrešević Đ., Sorić I. (2011). Students’ Emotions and Their Predictors in the Process of Self-Regulated Learning. Društvena Istraživanja Časopis Opća Društvena Pitanja.

[25] Andresen E.M., Malmgren J.A., Carter W.B., Patrick D.L. (1994). Screening for Depression in Well Older Adults: Evaluation of a Short Form of the CES-D. Am. J. Prev. Med..

[26] Eaton W.W., Smith C., Ybarra M., Muntaner C., Tien A., Maruish M.E. (2004). Center for Epidemiologic Studies Depression Scale: Review and revision (CESD and CESD-R). The Use of Psychological Testing for Treatment Planning and Outcomes Assessment.

[27] Cosco T.D., Prina M., Stubbs B., Wu Y.T. (2017). Reliability and validity of the center for epidemiologic studies depression scale in a population-based cohort of middle-aged U.S. adults. J. Nurs. Meas..

[28] Andresen E.M., Byers K., Friary J., Kosloski K., Montgomery R. (2013). Performance of the 10-item Center for Epidemiologic Studies Depression scale for caregiving research. SAGE Open Med..

[29] Hayes A.F., Montoya A.K., Rockwood N.J. (2017). The analysis of mechanisms and their contingencies: PROCESS versus structural equation modeling. Australas. Mark. J..

[30] Deslandes A., Moraes H., Ferreira C., Veiga H., Silveira H., Mouta R., Pompeu F.A.M.S., Coutinho E.S.F., Laks J. (2009). Exercise and Mental Health: Many Reasons to Move. Neuropsychobiology.

[31] Ren J., Xiao H. (2023). Exercise for mental well-being: Exploring neurobiological advances and intervention effects in depression. Life.

[32] Krishnan V., Nestler E.J. (2008). The molecular neurobiology of depression. Nature.

[33] Matei D., Trofin D., Iordan D.A., Onu I., Condurache I., Ionite C., Buculei I. (2023). The Endocannabinoid System and Physical Exercise. Int. J. Mol. Sci..

[34] Liu P.Z., Nusslock R. (2018). Exercise-Mediated Neurogenesis in the Hippocampus via BDNF. Front. Neurosci..

[35] Chen Z.Y., Jing D., Bath K.G., Ieraci A., Khan T., Siao C.J., Herrera D.G., Toth M., Yang C., Mcewen B.S. (2006). Genetic variant BDNF (Val66Met) polymorphism alters anxiety-related behavior. Science.

[36] Zahrt O.H., Crum A.J. (2017). Perceived physical activity and mortality: Evidence from three nationally representative US samples. Health Psychol..

